# When Do We Use Alternative Methods? Examination of the Factors Affecting the Use of Alternative Methods in Cochlear Implantation Surgery

**DOI:** 10.3390/jcm14186525

**Published:** 2025-09-17

**Authors:** Enver Can Öncül, Yüksel Olgun, Can Apaydın, Özden Savaş, Erdoğan Özgür, Enes Bilgin Türkmenoğlu, Enis Alpin Güneri

**Affiliations:** Department of Otorhinolaryngology, Dokuz Eylul University, 35000 Izmir, Türkiye

**Keywords:** cochlear implantation, cochlear hearing loss, inner ear malformations, hearing restoration

## Abstract

**Background/Objectives:** This study aimed to evaluate factors necessitating alternative techniques during cochlear implant (CI) surgery and to compare outcomes with standard procedures. **Methods:** Patients of all ages who underwent CI at our center between January 2003 and January 2024 were included. Alternative methods were defined as removal of external auditory canal/posterior canal wall, removal of ossicles to enhance surgical view, use of an endoscope for round window visualization, or similar approaches. **Results:** A total of 404 patients (mean age 13 ± 19.7 years) were analyzed. Preoperative imaging revealed inner ear anomalies in 44 patients (10.9%). Alternative methods were used in 41 patients (10.1%), including incus removal (*n* = 16), endoscopic assistance (*n* = 14), posterior canal wall removal (*n* = 4), incus buttress removal (*n* = 3), combined ossicle removal (*n* = 3), and one canal wall down mastoidectomy with fat graft obliteration and blind pouch closure. Alternative methods were significantly more frequent in patients with inner ear anomalies or additional otologic disease (*p* = 0.01 and *p* < 0.01, respectively), but not across age groups (*p* = 0.65). Partial electrode insertion occurred in 17 cases. Electrode insertion and complication rates were comparable between groups (*p* = 0.08 and *p* = 0.99, respectively). Bony cochleostomy was significantly more common in the alternative methods group (*p* = 0.01). **Conclusions:** Inner ear anomalies and additional otologic diseases may necessitate alternative CI techniques. These methods achieve electrode insertion and complication rates comparable to standard approaches, supporting their effectiveness in selected cases.

## 1. Introduction

Cochlear implantation (CI) has been widely used worldwide for the treatment of profound sensorineural hearing loss for more than sixty years [1,2]. Clinical results vary depending on various factors such as the onset and duration of hearing loss, the patient’s anatomical structure, neural physiology, neurodevelopmental disorders, the age at which the implant is applied, the level of psychosocial support, and the quality of postoperative rehabilitation. As a result of developments in these areas, the population undergoing implantation has expanded [3,4,5]. With the expansion of the population undergoing implantation, the number of CI applications to patient groups with additional otological diseases and inner ear anomalies has increased. As a result, the frequency of occurrence of previously uncommon situations in the intraoperative period has increased, and alternative methods that were not frequently applied before having begun to be applied more frequently.

Transmastoid posterior tympanotomy (PT) is the standard approach employed during CI. Recently, it has been suggested that eliminating the mastoid air cell system may lead to undesirable outcomes such as persistent negative middle ear pressure and tympanic membrane retractions, and technological advances in the era of minimally invasive surgery have led to the identification of alternative surgical techniques that avoid the mastoidectomy step of the procedure [6,7,8,9,10]. However, in some cases, it may be necessary to resort to an alternative method due to the limited anatomic view of the round window (RW) region when viewed through the PT aperture. The temporal bone is a complex structure that undergoes significant postnatal development and lateral growth; an average of 12 mm of growth has been reported directly between the sinodural angle and the RW between birth and adulthood [11,12]. Since the anterior and lateral located mastoid segment of the facial nerve is the leading cause of the narrow PT window and thus inadequate exposure of the RW region, we hypothesized that some structural and developmental factors may lead to an unfavorable position of the RW, resulting in a difficult surgical exposure through the PT.

This situation is well recognized by experienced surgeons but is rarely addressed in detail. A recent classification assessing accessibility to the RW membrane through the PT classified these complex cases as type III patients, in whom the RW membrane cannot be detected even after puncture of the RW niche [13]. Recent studies have emphasized the importance of preoperative high-resolution computed tomography (CT) findings and reported that radiological evaluations correlate with RW visibility [14,15]. Although the anatomical variations in the posterior tympanum and RW region have been described in detail, problems with surgical orientation, such as mistaking a wide subcochlear canaliculus for the RW niche, can lead to inadvertent implantation attempts, leading to failure and other significant complications [16]. Most studies advocate electrode placement via a membranous cochleostomy through the RW membrane, not only to ensure correct placement of the array in the scala tympani and minimize the risk of damage to residual hearing, but also to achieve a more ideal electrode position [17,18]. Bony cochleostomy, which is less preferred when there is limited access, can still be used; however, similar soft surgical techniques can be employed, and a suitable electrode position can be achieved with this approach [13].

However, anatomical variations and inner ear abnormalities may compel the surgeon to move beyond the standard technique and utilize alternative approaches.

This study aims to evaluate the factors that may necessitate the use of alternative techniques instead of the standard posterior tympanotomy during CI surgery, as well as to describe the surgical characteristics of these alternative methods.

## 2. Materials and Methods

### 2.1. Participants

Inclusion criteria for the study included patients of all age groups who underwent CI at our center between January 2003 and January 2024. All patients underwent high-resolution temporal bone computed tomography before surgery, and temporal magnetic resonance imaging was obtained for all pediatric cases. Surgical techniques were retrospectively evaluated from operative notes and classified into two groups: the PT group and the alternative technique (AT) group. The AT group included cases requiring removal of the posterior canal wall, endoscopic assistance, combined transcanal approaches, or cases performed via posterior tympanotomy but requiring maneuvers such as incus or posterior buttress removal. Exclusion criteria were having a cochlear implant in the same ear or having otological surgery in the same ear.

The study was approved by our institutional Research Ethics Committee (Decision No: 2022/23-04, dated 20 July 2022). The study was conducted in accordance with the guidelines of the 1975 Helsinki Declaration, as revised in 2013.

### 2.2. Procedures

All surgeries were performed at our center by two surgeons with more than 20 years of experience in CI surgery. All patients were admitted one day before surgery and discharged one day after surgery. All patients received single doses of prophylactic third-generation cephalosporin antibiotics, according to body weight, and were given Dexamethasone as a single dose of 0.3 mg/kg at induction. All operations were performed under general anesthesia. Patient demographics, additional otologic conditions (e.g., otitis media with effusion, chronic otitis media, otosclerosis, Ménière’s disease), the presence of inner ear anomalies according to the Sennaroğlu classification [19], and revision surgeries were evaluated as potential risk factors that might necessitate the use of an alternative CI method. Insertion characteristics (full vs. partial) and the rate of round window insertions were evaluated.

In this study, all intraoperative and postoperative events documented in operative notes or patient records were defined as “complications.” We did not distinguish between minor and major complications; rather, any event such as transient facial nerve twitching, wound seroma, wound infection, peripheral facial paralysis, postoperative nystagmus-vertigo, necrosis at the implant site, cerebrospinal fluid leak, or device-related problems was included under this definition. Complication rates were assessed in both groups. Facial nerve neuromonitoring was not routinely performed. However, a facial nerve neuromonitoring loop was applied to patients with inner ear anomalies detected during preoperative examinations.

### 2.3. Statistical Analysis

Statistical analyses were performed using IBM SPSS Statistics for Windows, Version 27.0 (IBM Corp., Armonk, NY, USA). Descriptive statistics for continuous variables were presented as mean, standard deviation, median, minimum, and maximum values, while categorical variables were expressed as numbers and percentages. The chi-square test or Fisher’s exact test was used to evaluate the distribution of categorical data between groups. A *p*-value of <0.05 was considered statistically significant.

## 3. Results

The mean age of the participants was 13 ± 19.7 years, with a median of 3 years (range: 1–78 years). Of these, 90 patients (22.3%) were in the adult age group, and 314 patients (77.7%) were in the pediatric age group. Regarding sex distribution, 209 patients (51.7%) were female and 195 (48.3%) were male.

Preoperative radiological examinations revealed that 44 of the 404 patients (10.9%) had an inner ear anomaly, whereas 360 patients (89.1%) did not. Based on the Sennaroğlu classification, the identified anomalies were as follows: large vestibular aqueduct (LVA) in 15 patients, incomplete partition type 2 (IP-2) in six, semicircular canal agenesis in six, incomplete partition type 1 (IP-1) in three, incomplete partition type 3 (IP-3) in three, internal acoustic canal dilation in three, internal acoustic canal dilation with cochlear aperture stenosis in two, vestibular duct dilation in two, cochlear nerve hypoplasia in two, cochlear hypoplasia in one, and semicircular canal agenesis with vestibular aqueduct enlargement in one patient.

Cases in which the standard PT approach was either not applied or modified during cochlear implantation were classified as alternative methods. These included removal of the posterior wall of the external auditory canal (EAC), use of an endoscope, including ossicular chain modifications such as incus buttress removal, combined ossicle removal, or canal wall down mastoidectomy with fat graft obliteration and blind pouch closure. Alternative methods were used in 41 patients (10.1%).

The distribution of AT was as follows: incus removal in 16 patients, endoscope-assisted CI in 14, posterior EAC wall removal in four, incus buttress removal in three, removal of both incus and stapes in two, removal of both incus and malleus in one, and canal wall down mastoidectomy combined with obliteration of the mastoid cavity and eustachian tube using an abdominal fat graft, followed by blind pouch closure of the EAC, in one patient.

Statistical analysis of alternative method use by age group, presence of inner ear anomaly, and presence of additional otologic disease showed no significant difference by age group (*p* = 0.65). However, the use of alternative methods was significantly higher in patients with an inner ear anomaly and in those with additional otologic disease (*p* = 0.01 and *p* < 0.01, respectively) (Table 1).

In 17 cases, electrode insertion was partial. Among these, three patients had an inner ear anomaly, two had otosclerosis, and one had a history of meningitis. Regarding the relationship between inner ear anomalies and electrode placement, partial insertion was observed in one patient with an IP-1 anomaly, one with a large vestibular aqueduct, and one with semicircular canal agenesis. In contrast, all other patients with inner ear anomalies achieved complete electrode insertion.

A total of eight patients had otosclerosis. Postoperative facial nerve stimulation was observed in two of these patients, and their complaints resolved following revision and reimplantation surgery. Facial stimulation was not observed in the group without otosclerosis.

ATs were compared with the standard approach in terms of electrode insertion, round window application rates, and complications. No statistically significant difference was found for electrode insertion or complication rates (*p* = 0.08 and *p* = 0.99, respectively). However, bony cochleostomy rates were significantly higher in the AT group compared to the standard method group (*p* = 0.01) (Table 2).

## 4. Discussion

CI is a surgical treatment for rehabilitating patients with severe to profound hearing loss and is indispensable for optimal language development, particularly in children [20]. The widespread implementation of neonatal hearing screening programs and the ability to diagnose hearing loss in the neonatal period have facilitated the early use of hearing rehabilitation tools such as hearing aids, as well as surgical interventions like CI [21].

The transmastoid–facial recess approach has been well established and standardized for routine CI. However, this standard technique may require modification to address preoperative or intraoperative challenges [22]. In some cases, it may be necessary to employ alternative methods due to restricted visualization of the round window from the facial recess, variations in the anatomy of the facial nerve or sinus tympani, intraoperative bleeding, or granulation tissue resulting from otitis media with effusion [14,15,23,24,25]. ATs include blind sac closure of the external auditory canal (EAC), removal of the posterior EAC wall, removal of middle ear ossicles to improve visualization of the round window, and endoscope-assisted implantation in selected clinical situations.

Endoscopic ear surgery has gained considerable popularity during the last decade, and this enthusiasm encouraged the otologists to use endoscopes during conventional and alternative CI surgeries [16,17,26,27,28]. In a series of 179 cases, Güneri et al. reported using endoscopic assistance to accurately identify the round window membrane in 14 cases (7.8%), with no complications observed. In all cases, electrodes were successfully advanced through the round window into the cochlea. Endoscopic identification of the round window membrane via the facial recess was deemed suitable in all cases without the need for bony cochleostomy or other more invasive alternatives. The primary advantage of endoscope-assisted CI is improved visualization, enabling a panoramic view of the round window region. This allows the surgeon to avoid cochleostomy and operate more safely with a lower complication risk [23].

In a series of 377 cases, Bae et al. reported an alternative method usage rate of 1.3%, consisting of posterior EAC wall removal in three patients (0.8%) and the endomeatal approach in two patients (0.5%). In cases where the round window membrane cannot be visualized from the facial recess, or in instances of severe cochlear rotation, temporary removal of the posterior EAC wall may be a viable method to expose the round window [29]. Their studies have examined the relationship between otitis media with effusion and the use of alternative methods. Cevizci et al., in their study comparing CI in patients with and without otitis media with effusion, reported that incus removal was required to visualize the round window in 14 of 105 patients (13.3%) with effusion. They noted that a narrow facial recess was present in three of 785 patients without effusion, but did not indicate whether an alternative method was applied [30]. Similarly, Pamuk et al. reported the need for incus removal in two of 169 patients with effusion (1.2%) [31].

In our study, alternative methods were used in 41 patients (10.1%): incus removal in 16, endoscope-assisted CI in 14, posterior EAC wall removal in four, incus buttress removal in three, incus and stapes removal in two, combined incus and malleus removal in one, and one case of canal wall down mastoidectomy combined with mastoid cavity and eustachian tube obliteration using an abdominal fat graft, followed by blind sac closure of the EAC. In our series, no cases of the endomeatal approach were performed. The decision to apply an alternative technique was primarily made intraoperatively, when RW exposure was deemed insufficient. In selected cases, preoperative imaging suggested possible need for AT, but the final choice was made during surgery. The bony cochleostomy rate was significantly higher in the alternative methods group (*p* = 0.01), whereas no significant difference was observed in complication rates (*p* = 0.99). From this perspective, although bony cochleostomy rates are higher when alternative methods are used, there is no difference in complication rates. Therefore, surgeons may consider alternative methods with greater confidence when standard exposure is inadequate.

In our cohort, the main reasons for employing alternative approaches included (i) restricted visualization of the round window due to a narrow or anatomically unfavorable facial recess, (ii) inner ear anomalies such as incomplete partition or vestibular aqueduct enlargement, (iii) additional otologic pathologies like otosclerosis, post-meningitic cochlear ossification, or chronic otitis media with effusion, and (iv) intraoperative challenges such as granulation tissue or excessive bleeding. In most cases, the decision to use an alternative method was made intraoperatively when adequate exposure of the round window could not be obtained despite a standard posterior tympanotomy. These findings highlight that alternative techniques are not routine choices, but are reserved for situations where standard exposure is inadequate.

The high prevalence of pediatric patients in our cohort can be partly attributed to national health policies and early detection programs in Turkey. Since 2003, the National Newborn Hearing Screening Program has been in place, with screening coverage reaching approximately 98% of newborns, and around 3 per 1000 infants confirmed with permanent congenital hearing loss. Infants who fail the screening are promptly referred for audiological diagnosis and rehabilitation. Moreover, the social health insurance system (SGK) covers the cost of cochlear implantation for young children under the criteria delineated in the national reimbursement guidelines, which likely contributes to early access to cochlear implantation and explains the predominance of pediatric CI cases in our center [32,33].

With the advancement of CI surgery and technological developments, this operation has begun to be applied to many patients with different etiologies. This situation may cause different difficulties and unpredictable results during surgery. Cochlear ossification or round window obliteration may develop in patients with advanced otosclerosis, which requires an extended cochleostomy. The electrodes may not be fully inserted into the cochlea, and some may remain outside. Despite the difficulties experienced during surgery, cochlear implantation can be successfully applied to these patients. Kabbara et al., in their study on 58 patients with advanced otosclerosis, divided the patients into three groups: the group with cochlear implants, the group with stapedotomy, and the group with cochlear implants after stapedotomy. In the study, better hearing results were obtained in the groups with cochlear implants [34]. Calvino et al., in their study on 239 patients, 22 of whom had otosclerosis, found that individuals with otosclerosis benefited significantly from the use of CIs. In addition, no significant difference was found between the two groups regarding the frequency of full electrode placement in the study [35]. In a meta-analysis of 21 studies conducted by Kondo et al., the rate of partial electrode insertion in CI cases with otosclerosis was 10% [36]. In our study, two of eight patients with otosclerosis (25%) had partial electrode insertion, which was high compared to the literature. This may be due to the difference in the degree and prevalence of otosclerosis between the studies, the onset of otosclerosis, and the time of CI application.

Another problem in otosclerosis patients is facial stimulation when the electrodes are activated after surgery. The increased incidence of facial stimulation with otosclerosis is due to the low impedance of the otosclerotic bone. It causes an increase in the conductivity between the basal turn of the cochlea and the facial nerve canal. Atanasova et al. In a study comparing 17 patients with otosclerosis and 21 patients without otosclerosis who underwent CI, the frequency of facial stimulation (13.6%) was slightly increased in patients with otosclerosis. However, it did not differ significantly from the frequency in the control group [37]. In a meta-analysis of Horn et al., which included 5694 patients, the overall facial stimulation rate was 5.6%. This rate was found to be 26% in cases with otosclerosis [38]. The rate in this meta-analysis is consistent with our study. In our case series, postoperative facial nerve stimulation was observed in two of eight patients with otosclerosis, and the complaints of the patients regressed as a result of revision and reimplantation surgery. Facial stimulation was not observed in the non-otosclerosis group. Advanced processes such as hyalinization, cavity formation, changes in the electrical conductivity of the bone, and the decrease in the distance between the electrode and the facial nerve cause facial nerve stimulation with auditory stimuli. It can be managed with methods such as distal electrode deactivation or revision surgery.

Inner ear anomalies are found in approximately 20% of individuals with congenital sensorineural hearing loss. Sennaroğlu et al. emphasized that accurate classification of inner ear anomalies improves CI outcome [19]. Daneshi et al., in a study of 107 patients, reported satisfactory results for CI in those with inner ear anomalies [39]. Karamert et al. noted post-CI performance differences in these patients but stressed the importance of CI in developing communication skills, with the best verbal scores observed in patients with LVA, IP-2, and cochlear hypoplasia type 2 [40]. Buchman et al., in their analysis of surgical and audiological outcomes in 28 children with inner ear malformations, concluded that CI can be performed safely in this group [41].

Farhood et al. similarly found successful CI outcomes in patients with inner ear anomalies, with complete electrode insertion achieved in 77.7% of cases according to the Sennaroğlu classification [19,42]. Vashist et al. reported complete electrode insertion in all patients with inner ear anomalies who experienced intraoperative CSF leakage, although the sample size was small. They did not report electrode insertion status in cases without CSF leakage [43]. Isaiah et al. reported partial electrode insertion in 6.4% of cases with inner ear anomalies, compared to complete insertion in 3.8% of cases, noting technical challenges during electrode placement [2]. Melo et al. reported partial electrode insertion in 3.8% of patients with inner ear anomalies [44].

While prior studies have examined the association between inner ear anomalies and electrode insertion, few have explored their relationship with the need for alternative methods. In our study, a statistically significant increase in the use of alternative methods was observed among patients with inner ear anomalies (*p* = 0.01). Nevertheless, electrode insertion rates in these patients, even when using alternative techniques, were comparable to those achieved with standard methods (*p* = 0.08).

This study spans a period of 20 years, during which surgical techniques and cochlear implant technology have evolved. Both senior surgeons contributed to all procedures throughout this timeframe, which minimizes the impact of surgeon-dependent variability. However, differences between the early and late periods were not formally analyzed, and evolving technology and surgical experience may have influenced outcomes. This represents a limitation of the present study.

Despite primarily relying on HRCT/MRI and surgical expertise in our case series, recent advances in otological planning software such as tablet-based tools and OTOPLAN imaging have emerged as valuable adjuncts in cochlear implant surgery [45,46]. Tablet-based software has demonstrated high inter- and intra-rater reliability in measuring cochlear duct length, which is critical for determining appropriate electrode array insertion depth [47]. Similarly, OTOPLAN-based reconstructions have enabled accurate identification of cochlear trajectory, particularly in cases with anatomical alterations such as post-meningitic ossification or advanced otosclerosis [48,49].

These imaging tools enhance the surgeon’s preoperative anatomical orientation by providing patient-specific spatial reconstructions, which could reduce intraoperative uncertainty and guide the selection of surgical approach or alternative techniques.

### 4.1. Limitations

The main limitations of this study include its retrospective design, the single-center setting, and the long study period spanning more than 20 years, during which surgical techniques and technologies have evolved. In addition, the heterogeneity of the patient population and the relatively small number of cases requiring alternative methods limit the generalizability of our findings.

Another limitation of our study is the lack of audiological outcome data comparing standard and alternative approaches. Since our primary objective was to evaluate intraoperative and perioperative surgical factors, detailed postoperative audiological measures such as aided thresholds, CAP (Categories of Auditory Performance)/SIR (Speech Intelligibility Rating) scores, or speech perception tests were not systematically analyzed. Although several studies in the literature have reported favorable hearing and speech outcomes following cochlear implantation in patients with inner ear anomalies or additional otologic diseases, our data do not allow for direct comparison in this regard. Future prospective studies integrating both surgical and audiological outcomes would provide more comprehensive insights into the overall effectiveness of alternative techniques.

### 4.2. Clinical Utility

From a clinical perspective, our results support the notion that alternative techniques are reliable and safe strategies in anatomically challenging cases. Recognizing these factors preoperatively may help surgeons anticipate intraoperative challenges, reduce complications, and improve surgical outcomes.

### 4.3. Future Prospects

Future studies should prospectively evaluate the role of endoscopic approaches, 3D planning software, and artificial intelligence–based systems in predicting difficult anatomical cases. Such tools may improve preoperative decision-making and help surgeons anticipate the need for alternative techniques.

In line with recent advances, artificial intelligence (AI) systems are gaining importance in surgical decision-making, including in the field of cochlear implantation. These tools have the potential to analyze preoperative radiological studies together with patient-specific clinical parameters, thereby supporting surgeons in identifying the most appropriate surgical approach. A preliminary study by Portelli et al. has recently demonstrated the feasibility of using ChatGPT (https://chatgpt.com, accessed on 31 January 2025) and Microsoft Copilot (https://copilot.microsoft.com, accessed on 31 January 2025) to aid in cochlear implant side selection, underlining the applicability and clinical relevance of AI-based systems in this domain [50]. While still in early stages, such approaches may become valuable adjuncts to preoperative planning, especially in complex anatomical cases.

## 5. Conclusions

The presence of inner ear anomalies or additional otologic diseases during cochlear implantation may necessitate the use of alternative surgical methods. As the indications for CI expand with time, surgeons must be familiar with alternative techniques in order to achieve successful implantation. When used in anatomically challenging cases, alternative techniques provide outcomes comparable to standard methods, underscoring their value as reliable options in modern clinical practice CI surgery.

## Figures and Tables

**Table 1:** Evaluation of alternative method use according to age group, presence of inner ear anomalies and additional otological diseases.

		Alternative Method		Total	*p* Value *
		Yes	No		
Age Group	Pediatric	33	281	314	0.65
	Adult	8	82	90	
Inner Ear Anomaly	Yes	13	31	44	**0.01**
	No	28	332	360	
Additional Otological Disease	Yes	21	104	125	**<0.01**
	No	20	259	279	

*p* < 0.05 significance level; * Chi-Square Tests.

**Table 2:** Comparison of standard and alternative methods by electrode insertion, bony cochleostomy, and complication rates.

		Alternative Method		Total	*p* Value *
		Yes	No		
Electrode Insertion	Full	37	350	387	0.08
	Partial	4	13	17	
Round Window (RW)/Bony cochleostomy (COC)	Bony cochleostomy	29	153	182	**0.01**
	Round window	12	210	222	
Complication	Yes	5	44	49	0.99
	No	36	319	355	

*p* < 0.05 significance level, * Chi-Square Tests.

## Data Availability

Due to privacy restrictions all data are stored at the researcher’s institution. Qualified researchers will be able to gain access via application at the corresponding author.
